# Chronic Heart Failure: We Are Fighting the Battle, but Are We Winning the War?

**DOI:** 10.6064/2012/279731

**Published:** 2012-12-20

**Authors:** John J. Atherton

**Affiliations:** ^1^Cardiology Department, Royal Brisbane and Women's Hospital, Butterfield Street, Herston, QLD 4006, Australia; ^2^School of Medicine, University of Queensland, Brisbane, QLD 4006, Australia

## Abstract

Heart failure represents an end-stage phenotype of a number of cardiovascular diseases and is generally associated with a poor prognosis. A number of organized battles fought over the last two to three decades have resulted in considerable advances in treatment including the use of drugs that interfere with neurohormonal activation and device-based therapies such as implantable cardioverter defibrillators and cardiac resynchronization therapy. Despite this, the prevalence of heart failure continues to rise related to both the aging population and better survival in patients with cardiovascular disease. Registries have identified treatment gaps and variation in the application of evidenced-based practice, including the use of echocardiography and prescribing of disease-modifying drugs. Quality initiatives often coupled with multidisciplinary, heart failure disease management promote self-care and minimize variation in the application of evidenced-based practice leading to better long-term clinical outcomes. However, to address the rising prevalence of heart failure and win the war, we must also turn our attention to disease prevention. A combined approach is required that includes public health measures applied at a population level and screening strategies to identify individuals at high risk of developing heart failure in the future.

## 1. Introduction

Heart failure is a clinical syndrome that represents the end-stage phenotype of a number of different cardiovascular diseases. However, regardless of the underlying cause, the prognosis is generally poor once a patient develops symptoms or signs of heart failure. There has been considerable progress in management including the use of drugs that interfere with neurohormonal activation, device therapy in selected patients, and multidisciplinary disease management. This at least partly explains the reductions in heart failure mortality seen in population-based studies over the last two decades; however, the overall epidemiological impact has been modest. This paper will provide an overview of the epidemiology of heart failure and outline our continued efforts to fight the battle focusing on management strategies that have been shown to modify disease progression. I will then consider whether or not we are winning the war and suggest that efforts to minimize variation in care should continue coupled with increasing emphasis on heart failure prevention.

## 2. Epidemiology of Heart Failure 

Heart failure is one of the few cardiovascular diseases whose prevalence continues to rise, largely related to the aging population accompanied by improved survival in patients with heart failure and following myocardial infarction [1, 2]. Incident heart failure hospitalization rates reached a peak in the 1990s which then plateaued and in most studies now appear to be falling [3–12]. However, incident rates based upon community cohorts have shown little change [13–15], with a young adult having a 20–30% lifetime chance of developing heart failure [16, 17]. Consequently, age-adjusted prevalence rates continue to rise with heart failure imposing a substantial human and economic burden in both developed and emerging economies. In developed economies, direct costs related to heart failure account for 1-2% of the total healthcare expenditure, largely accounted for by costs incurred during hospitalization [18–20].

The prognosis of patients with heart failure is generally poor with frequent admissions to hospital throughout the disease trajectory and median survivals of 3–5 years [12–14, 17, 23–28]. There have been consistent reductions in both short-term and long-term mortality over the last two decades, which probably reflects increasing utilization of evidence-based therapies and better management of cardiovascular risk factors [3–7, 10–15, 25, 28–34]. Most of the improvement in outcomes has been in younger patients with left ventricular (LV) systolic dysfunction (LVSD), which is perhaps not surprising given that the evidence-base for disease modifying therapies is strongest in this population [4, 6, 13, 28, 35]. A recent individual patient data meta-analysis demonstrated better survival in patients with preserved LV ejection fraction (LVEF) compared with patients with reduced LVEF following adjustment for age and comorbidity [36]. These findings are consistent with a meta-analysis of prospective observational studies where LV function had been assessed in almost all patients; the long-term mortality in patients with heart failure associated with preserved LVEF was approximately one half that of patients with reduced LVEF [37]. Nonetheless, once an individual develops symptomatic heart failure, their outcome is generally poor regardless of underlying LV function [35, 38–43]. Furthermore, heart failure patients often suffer associated comorbidities, which complicate management and are associated with increased hospitalization rates and poorer survival [6, 12, 44, 45]. Indeed, this largely explains why most rehospitalizations are due to noncardiovascular diseases [45].

## 3. Modifying the Disease Trajectory: Evidenced-Based Management

Despite heart failure representing an end-stage phenotype of different cardiovascular diseases, numerous large-scale, randomized, and placebo-controlled studies have extended our mechanistic understanding of this syndrome. Most of these studies have been conducted in patients with moderate to severe LVSD regardless of the underlying cause. Broadly speaking, the therapeutic approaches can be divided into pharmacological therapies, device-based therapies, and coordinated disease management [46–51].

### 3.1. Pharmacological Therapies

Treatments primarily focused on managing congestion and hemodynamic derangements play an important role in treating decompensated heart failure with loop diuretics remaining the most effective pharmacological approach to manage congestion. However, these approaches have a limited effect on long-term clinical outcomes. Over the last three decades, a number of multicenter studies conducted in patients with symptomatic and asymptomatic LVSD have highlighted the role of neurohormonal activation on disease progression (see Table 1) [21, 52–65]. 

Angiotensin converting enzyme (ACE) inhibitors improve symptoms, reduce hospitalization, and prolong survival in patients with symptomatic heart failure associated with moderate to severe LVSD both early following myocardial infarction and in the setting of chronic heart failure regardless of the underlying etiology [52, 53, 55, 58, 65]. Subsequent studies have demonstrated similar benefits from angiotensin receptor blockers in the setting of heart failure associated with LVSD, either as an alternative to ACE inhibitors [54, 66], in patients who were previously intolerant of ACE inhibitors [67], or in addition to ACE inhibitor therapy [68]. Furthermore, the combined endpoint of death and heart failure hospitalization was reduced by the addition of an ACE inhibitor to standard care in patients with asymptomatic LVSD [22], which translated to improved long-term survival [21]. 

However, despite the well-established benefit from blockade of the renin-angiotensin system, the long-term cumulative mortality rates remained high reflecting ongoing disease progression, often associated with progressive adverse LV remodeling. Subsequent large, placebo-controlled studies emphasized the need for a combined approach to neurohormonal blockade. The addition of a beta blocker reduced mortality by 34-35% and decreased heart failure hospitalization in chronic heart failure patients with underlying moderate to severe LVSD on top of standard therapy which included an ACE inhibitor and diuretic in the majority of patients [59–61].

Given the established mechanistic role of aldosterone in the pathogenesis of cardiovascular disease and that aldosterone synthesis is only modestly suppressed by chronic renin-angiotensin system blockade [69, 70], studies have explored the therapeutic role of mineralocorticoid receptor antagonists. Consistent mortality reductions have been demonstrated on top of ACE inhibitor therapy (with or without additional beta blockade) in the setting of LVSD in both chronic heart failure and early following myocardial infarction [62–64]. Concerns have been raised regarding the broader application of dual blockade of the renin-angiotensin-aldosterone system [54], especially in the real-world setting without careful attention to patient selection and electrolyte monitoring [71]. In the Aliskiren Trial in Type 2 Diabetes Using Cardio-Renal Endpoints (ALTITUDE) study, the combination of the direct renin inhibitor, aliskiren, and either an ACE inhibitor or an angiotensin receptor blocker was associated with an increased risk of renal impairment, hyperkalemia, and hypotension. This led the US Food and Drug Administration to recommend a new contraindication against the use of this combination in diabetics and a warning to avoid the combination in patients with moderate to severe renal impairment (see http://www.fda.gov/drugs/drugsafety/ucm300889.htm). Furthermore, a population-based study conducted in Canada reported increased hospitalizations and deaths related to hyperkalemia, associated with increased rates of prescribing spironolactone to heart failure patients treated with ACE inhibitors following the publication of the Randomized Aldactone Evaluation Study (RALES) [71]. However, a more recent study conducted in Scotland suggests that regular monitoring of serum potassium and renal function allowed early identification of mild hyperkalemia and appropriate dose adjustment. Increased prescribing of spironolactone occurred in parallel with increased measurements of serum creatinine and potassium after the publication of the RALES results and did not translate to increased hospitalizations for hyperkalemia. Indeed, there was a trend of reduced rates of severe hyperkalemia over time [72].

Additional studies have reported beneficial effects of adding various 2nd-line agents in selected patients including ivabradine (in patients in sinus rhythm of 70 beats per minute or greater despite optimal medical therapy), polyunsaturated fatty acids, hydralazine and nitrates, digoxin, and intravenous iron therapy [49, 50, 73–77].

### 3.2. Device-Based Therapies

Combined neurohormonal blockade has been shown to reduce both heart failure related and sudden (presumed arrhythmic) deaths and, in some cases, reversal of the adverse LV remodeling process. However, progressive adverse remodeling continues despite optimal pharmacological therapy in a large proportion of patients. Implantable cardioverter defibrillator therapy reduces the risk of arrhythmic death, which translates to improved survival in patients with severe LVSD associated with symptomatic chronic heart failure or following previous myocardial infarction (see Table 1) [78, 79]. However, unlike pharmacological treatments where costs are distributed over time, the upfront costs associated with device therapies impact on cost effectiveness, which is sensitive to the projected time horizon [80–83]. Therefore, in patients where the short-to-medium-term outcomes are driven by age and associated comorbidities, these treatments are unlikely to be clinically effective and cost effective. Nonetheless, provided patients are carefully selected, the use of implantable cardioverter defibrillators for the primary prevention of sudden cardiac death in heart failure patients with severe LVSD despite optimal medical therapy appears to be cost effective [84].

Approximately 20–30% of patients with heart failure due to LVSD have abnormal intraventricular conduction manifested by a prolonged QRS duration on the 12-lead electrocardiogram [85]. Short-term studies have demonstrated that resynchronizing ventricular contraction with biventricular pacing improves symptoms, exercise capacity, and quality of life [86–88], with these benefits maintained for at least 12 months [89, 90]. Subsequent large, and randomized, controlled trials demonstrated improved long-term survival and reduced heart failure hospitalizations with cardiac resynchronization therapy (CRT) in patients with symptomatic heart failure due to severe LVSD associated with ventricular dyssynchrony (largely based upon having a prolonged QRS duration on the 12-lead electrocardiogram) despite optimal medical therapy (see Table 1) [91–95]. These benefits were seen using either isolated biventricular pacing (CRT only device) [95] or when combined with an implantable cardioverter defibrillator (CRT-D device) [92, 94]. Furthermore, recent studies have clearly demonstrated that biventricular pacing reduces morbidity and mortality on top of the reduction in sudden death associated with using an implantable cardioverter defibrillator [92, 93].

### 3.3. Coordinated Disease Management

The management of chronic heart failure includes lifestyle adjustment, initiation and uptitration of medical therapy, and selective consideration of device therapy. These cases are often diagnosed following an acute episode of decompensation with 25–40% of patients admitted to hospital with acute decompensated heart failure having no prior documented history of heart failure [96–98]. The patients are generally acutely unwell and during their relatively brief hospital stay are informed that they have a serious illness that requires substantial adjustments to their usual lifestyle; commenced on a number of new drugs with an average of 10-11 drugs being prescribed on discharge; that they need to undertake lifelong disease monitoring; and see their primary care practitioner for further prescriptions and electrolyte monitoring. It is therefore hardly surprising that following discharge from hospital, up to one in four patients are readmitted within 30 days [26, 27] and 30–40% of patients will die or be readmitted within 60–90 days [99]. Furthermore, most of these hospital readmissions are potentially avoidable [100].

A large body of evidence supports the use of coordinated, multidisciplinary models of care usually led by heart failure nurses to support the transition of care from the acute hospital setting to community health providers. These services have been shown to prolong survival, reduce hospitalizations, and improve quality of life [101–105]. Furthermore, they are cost effective provided that appropriate patients are selected [105, 106]. Key features of effective heart failure programs include involving a multidisciplinary team with a cardiac nurse supported by a cardiologist; in-hospital referral with discharge planning; developing an individualized healthcare plan that incorporates patient and family education and counseling; promoting self-care behaviors including daily weighing and adherence to specific lifestyle measures and medications; medication optimization; ongoing follow-up and disease monitoring; and providing a mechanism to allow patient-initiated access to advice [107, 108].

## 4. Process Measures of Healthcare: Are We Applying the Evidence?

Whilst there is a rich body of evidence supporting the safety and clinical efficacy of pharmacological and device-based therapies in heart failure, the key question is whether this translates to clinical effectiveness in a real-world population. Recent registries and population-based cohorts of patients admitted to hospital with acute decompensated heart failure have highlighted treatment gaps and considerable variation in the quality of care provided in different hospitals [97, 98, 109–115]. Similar variation has been reported in the ambulatory care setting [116–120]. Despite all heart failure guidelines advocating the critical role of echocardiography to confirm the diagnosis, identify the underlying mechanism of heart failure, and guide therapy, 10–25% of patients admitted to hospital with a primary diagnosis of heart failure have no documented record that LV function has been assessed [97, 98, 115, 121, 122]. This is concerning given that failure to perform an echocardiogram in heart failure patients is associated with poorer long-term survival and under-prescribing of evidenced-based therapies [123]. 

Whilst the rates of prescribing disease modifying agents in patients admitted to hospital with heart failure have improved over the last decade, recent registries conducted in Europe, the Asia-Pacific region, and the USA still report discharge prescription rates of 63–80% for ACE inhibitors or angiotensin receptor blockers, 41–74% for beta blockers, and 20–48% for aldosterone antagonists [97, 98, 115, 122]. Furthermore, these drugs are generally prescribed at doses much lower than those achieved in the clinical trials (see Table 2) [124–137]. Importantly, improved adherence to guidelines is associated with better long-term clinical outcomes highlighting the need to minimize variation in process measures of healthcare to improve overall clinical outcomes [119, 138].

## 5. Why Are Not We Applying the Evidence?

Reasons for the gaps in treatment provided in real-world practice compared to clinical trials may include the limitation of using administrative datasets to code heart failure patients, the different populations being treated, the limitation of assessing treatment in hospital following an acute decompensation, and the level of care provided.

Administrative datasets rely on appropriate documentation by the treating clinicians and accurate coding by administrative staff. A number of studies have evaluated the accuracy of administrative coding demonstrating high positive predictive accuracy compared with independent chart abstraction or objective scoring systems such as the Boston score criteria [141–143]. Whilst this may not apply in all jurisdictions and does not exclude the possibility that heart failure patients are being incorrectly coded with an alternative diagnosis, it nonetheless indicates that inaccurate coding is unlikely to explain the variation in care observed between real-world registries and clinical trials.

Patients treated in clinical trials are on average over a decade younger than the patients we see in our clinical practice. Furthermore, the clinical trials which guide our practice either exclusively or largely enrolled patients with moderate to severe LVSD; yet up to half the patients enrolled in heart failure registries and population-based cohorts have preserved LVEF [35, 97, 98, 115, 122, 144–147]. The enrollment criteria for most clinical trials generally exclude patients with major comorbidities, documented contra-indications, poor compliance, and prior drug intolerance; yet these patients dominate our clinical practice. Many clinical trials also included a prerandomization, run-in phase to identify patients who are unlikely to tolerate uptitration of medical therapy. Indeed, over 90% of patients enrolled in heart failure registries would not have met the enrollment criteria for the clinical trials which guide our practice [148]; yet we attempt to apply this evidence to all patients. However, this is unlikely to fully explain the wide variation in care both within and between different countries. Indeed, even when ideal clinical indicators are crafted to match the inclusion and exclusion criteria used in the clinical trials, substantial treatment gaps remain [98, 110–113, 148].

Hospital-based registries generally report in-hospital metrics to measure quality performance. Whilst performance of echocardiography and discharge prescription of ACE inhibitors or angiotensin receptor blockers are well-accepted inpatient quality measures of healthcare, discharge prescription of beta blockers was not included in the Joint Commission on Accreditation of Healthcare Organizations (JCAHO) heart failure performance indicators (see http://www.jointcommission.org/specifications_manual_for_national_hospital_inpatient_quality_measures.aspx). However, few patients will have new therapies commenced in the first few months following hospital discharge [113]. Indeed, most patients can be safely commenced on beta blockers prior to discharge from hospital and are then more likely to receive these drugs compared with patients where the decision to start beta blockers is deferred to their first outpatient visit. Furthermore, provided patients are appropriately selected, this practice has no adverse effect on either hospital length of stay or subsequent treatment withdrawal rates [149].

Finally, it is likely that the model of care provided in real-world practice differs substantially from that provided in clinical trials. In the real world, clinical practice will be constrained by variable clinician awareness of the guidelines, a number of external barriers, and clinical inertia. Conversely, in clinical trials, investigators are generally familiar with the trial protocol and background evidence and patients care is supported by research nurses who assist in clinical assessments at outpatient visits, regularly document pharmacotherapy, monitor compliance, provide a point-of-contact for trial participants, and apply protocol-driven, forced drug uptitration. Indeed, in many ways coordinated, multidisciplinary heart failure disease management brings us closer to the models of care provided in the clinical trials on which we base our practice.

## 6. Where to Now?

Over the last two to three decades, considerable therapeutic gains have been made in the management of heart failure associated with LVSD with in excess of 50% cumulative reductions in mortality and associated reductions in hospitalizations in the randomized, controlled trials (see Table 1) [46–48]. However, the epidemiological impacts have been relatively modest given that these approaches have been variably applied to a proportion of heart failure patients at a relatively late stage in their disease trajectory. Therefore, whilst we should continue to implement best practice guided by the clinical trials, we also need to focus on early disease detection and preventive strategies.

### 6.1. Heart Failure Management

Substantial gains will be realized simply by implementing the treatments we know to be effective. A variety of strategies can be used to close the treatment gaps including reminder systems, timely feedback, and alternative models of care [150, 151]. In The Registry to Improve the Use of Evidence-Based Heart Failure Therapies in the Outpatient Setting (IMPROVE HF) which included 34,810 patients with reduced LVEF, a number of quality initiatives including clinical decision support tools, reminder systems, chart audits with timely feedback, benchmarked reports, and educational outreach were implemented across 167 outpatient practices in the USA. This resulted in significant improvement in five of seven quality indicators including increases in appropriate beta blocker prescribing (86% to 92%), mineralocorticoid receptor antagonist prescribing (35% to 60%), CRT (37% to 66%), and implantable cardioverter defibrillators (50% to 78%) [152]. Furthermore, these quality measures were associated with improved long-term survival [138]. Similar quality improvement initiatives have been successfully trialed in the acute hospital setting [121, 153]. In the Organized Program to Initiate Lifesaving Treatment in Hospitalized Patients With Heart Failure (OPTIMIZE-HF), real-time feedback of benchmarked reports resulted in increased complete discharge instructions (47% to 67%), smoking cessation counseling (48% to 76%), assessment of LV function (89% to 92%), and beta blocker prescribing (76% to 86%). Furthermore, use of clinical decision support tools was associated with improved process measures of healthcare and lower adjusted in-hospital mortality [121].

**Figure 1:**
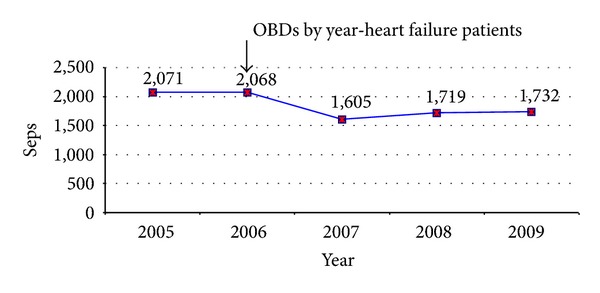
Number of occupied bed days per calendar year from 2005 to 2009 for patients admitted to the Royal Brisbane and Women's Hospital with a primary diagnosis of heart failure (ICD codes 150.0 to 150.9). The arrow indicates the introduction of the multidisciplinary heart failure service. OBDs = occupied bed days.

In many centers, multidisciplinary heart failure disease management programs which incorporate a number of these quality improvement measures have been implemented. In our state of Queensland, which has a population of 4.5 million and land mass of 1.7 million square kilometers, we received government funding to appoint heart failure nurses in all 22 health districts. We developed a multidisciplinary steering committee to oversee the implementation and monitoring of these services and continue to hold an annual 2-day specialized heart failure course to support the training of nursing and allied health staff [154]. Following the implementation of the heart failure service in our hospital, we observed a reduction in occupied bed days for heart failure, which was sustained over the next three years (see Figure 1). Whilst we cannot necessarily imply cause-and-effect, it nonetheless suggests that the beneficial effects observed in clinical trials translated to clinical effectiveness in our practice.

Over 90% of newly referred heart failure patients with LVSD now receive ACE inhibitors and beta blockers in our service, however only one quarter of patients who are not on target doses of therapy when they leave hospital will achieve target doses by 6 months (unpublished data). Our local experience matches that seen in most studies that report prescribing patterns in clinical practice (see Table 2) [124–137, 155], but falls well below that seen in the clinical trials where 66–80% of patients achieved target doses of beta blockers. Heart failure services that involve nurses in protocol-driven uptitration of medical therapy are more likely to achieve target doses [133, 135, 156, 157]; however, these services are not universally available and follow-up arrangements may vary according to clinician preference and patient choice. We implemented a statewide heart failure medication uptitration form that clearly indicates who is responsible for uptitrating therapy and provides monitoring and trouble-shooting guidelines. We observed that patients who received a written uptitration plan were more than two-fold likely to achieve target doses by 6 months [158]. We are currently evaluating whether providing incentive payments to heart failure services for completing heart failure uptitration forms will further improve prescribing patterns.

The role of supplementary monitoring strategies to guide therapy and support disease management including telemonitoring and biomarkers remains the active area of investigation. Noninvasive telemonitoring has shown promise [159, 160]; however, recent large studies have been less favorable, highlighting the difficulties involved in incorporating diagnostic monitoring strategies into a continually evolving standardized model of care [161, 162] and the limited sensitivity of symptoms and weight change to identify heart failure exacerbations [163–165]. Meta-analyses of randomized, controlled trials have demonstrated that using B-type natriuretic peptides to monitor heart failure was associated with reduced mortality [166, 167], with this benefit at least partly achieved by using higher doses of disease-modifying drugs [166]. There was however no effect on hospitalization and the results of individual studies have been conflicting with none adequately powered for mortality. Furthermore, most of the studies to date have measured natriuretic peptides on an “occasional basis” (often 1-2 monthly). Given that the natriuretic peptides have a relatively short half-life measured in hours, it is likely that more frequent monitoring is required to guide therapy, especially in recently decompensated patients. Invasive monitoring using implantable devices to either directly or indirectly assess volume status allows for continuous monitoring and has been shown to identify patients at increased risk of heart failure hospitalization [168–172]. However, the low specificity of intrathoracic impedance to predict heart failure hospitalization [173] resulted in increased utilization of healthcare resources and higher rates of hospitalization in a recent randomized, controlled trial [174]. Conversely, a recent study using a wireless hemodynamic monitor demonstrated a large reduction in hospitalization in heart failure patients randomized to having daily measurement of pulmonary arterial pressure to guide their management [175]. Whilst further studies are ongoing, the clinical utility of monitoring strategies is entirely dependent on how the information provided will change management. Clinical algorithms will need to be validated and should ideally empower the patients and their family in the long-term management of their chronic disease.

Finally, most of the therapeutic gains made with drugs and device therapy have been in heart failure due to LVSD. Studies conducted in patients with preserved LVEF have been disappointing, which probably at least partly relates to the difficulty in clearly defining this population [176–178]. Further studies are awaited including the Treatment Of Preserved Cardiac Function Heart Failure with an Aldosterone Antagonist (TOPCAT) trial which is evaluating the role of mineralocorticoid receptor antagonists [179]. Comorbidities such as anemia, lung disease, kidney disease, diabetes, and depression represent major drivers of mortality, hospitalization and quality of life in heart failure patients [44, 45]. Future studies will need to improve our understanding of whether directly treating comorbidities not only improves clinical outcomes, but also modifies heart failure progression [45].

### 6.2. Heart Failure Prevention

Whilst efforts to improve the quality of care and clinical outcomes of heart failure patients should continue, this will have a limited epidemiological impact on the human and economic burden imposed by heart failure in our society. Unless we also focus on early disease detection and prevention, we are in effect chipping away at the tip of the iceberg as incident cases of heart failure continue to emerge. The American College of Cardiology Foundation/American Heart Association (ACCF/AHA) staging classification emphasizes the need for disease prevention akin to the approach applied in cancer prevention [139]. This classification describes presymptomatic phases where subjects are at increased risk of developing heart failure in the future which includes hypertension, atherosclerotic disease and diabetes (referred to as Stage A), and asymptomatic structural heart disease such as LVSD, LV hypertrophy, and valvular heart disease (referred to as Stage B), followed by the development of symptomatic heart failure (Stage C) and refractory heart failure despite optimized therapy (Stage D). Indeed, in a community-based, cross-sectional sample of adults aged 45 years and older, for every subject with symptomatic heart failure, there were three to four subjects with ACCF/AHA Stage A or B presymptomatic heart failure [180]. 

To address the presymptomatic phase of heart failure, a combined approach is required that encompasses both public health measures applied at a population level and identification and management of individuals at high risk of developing heart failure in the future. Public health measures should focus on environmental contributors with the ultimate aim being to encourage and facilitate healthy lifestyles through education, legislation, and urban planning [181]. Smoking cessation, blood pressure lowering, and LDL-cholesterol lowering reduce cardiovascular events and long-term mortality [182–184], with greater benefits in high risk individuals [185]. Furthermore, pharmacological approaches are likely to be more cost-effective if we can target them to individuals with preclinical structural cardiovascular disease who are at increased risk of future cardiovascular events including heart failure. 

The best example of this is asymptomatic LVSD which is associated with a five-fold increased risk of developing heart failure with over half dying (most from cardiovascular causes) before they present with clinical heart failure [186]. This represents a missed opportunity to administer preventive therapy. Provided we can detect individuals with asymptomatic LVSD, there are evidence-based treatments available including ACE inhibitors, which have been shown to reduce the risk of developing heart failure and improve long-term survival compared with waiting for individuals to develop symptoms or signs of heart failure before starting treatment (see Table 3) [21, 22]. Guidelines also advocate for the use of beta blockers in such patients, especially if there is a history of previous myocardial infarction [46, 48, 51]. Implantable cardioverter defibrillator therapy may be considered to reduce the risk of sudden arrhythmic death if there is persisting severe LVSD despite optimal medical therapy [51]. Identification of asymptomatic LVSD may also lead to further diagnostic workup for underlying coronary artery disease, which would lead to additional preventive therapies if detected.

**Figure 2:**
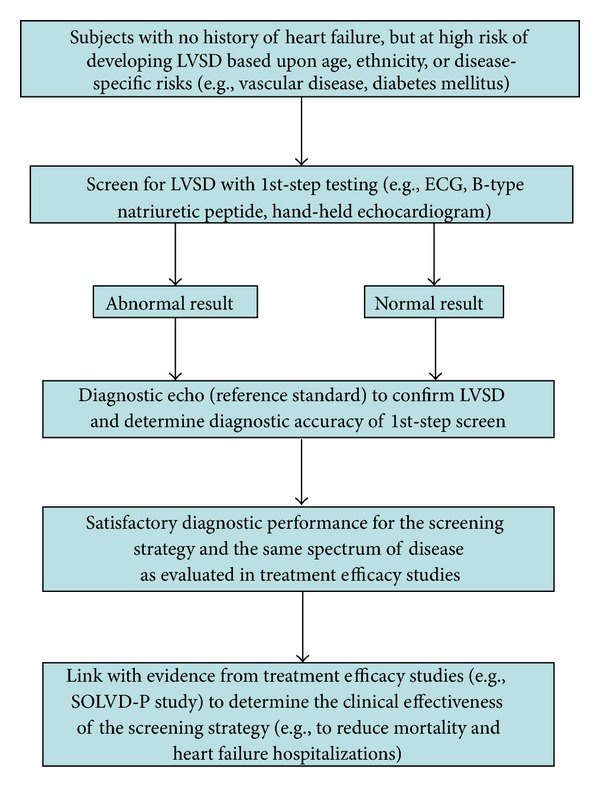
In situations where we can reliably detect disease (e.g., left ventricular systolic dysfunction) in properly conducted diagnostic screening studies, coupled with high-level evidence (preferably from randomized, controlled trials) that we can improve clinical outcomes in individuals with the same disease in treatment efficacy studies, then we can link the two to determine the clinical effectiveness of a screening strategy. LVSD = left ventricular systolic dysfunction; ECG = electrocardiogram; SOLVD-P = studies of left ventricular dysfunction-prevention [21, 22].

If we can accurately detect LVSD, it would seem reasonable to link this with the evidence from treatment efficacy studies conducted in subjects with asymptomatic LVSD to infer the clinical effectiveness of a screening strategy, provided the subjects detected through screening represent the same spectrum of disease as the patients enrolled in the clinical trials (see Figure 2) [140, 187]. However, given the limited availability of echocardiography and trained imaging staff, and the overall low prevalence of LVSD in the general community [40, 186, 188–195], a number of studies have evaluated the diagnostic accuracy of other methods to initially screen subjects including clinical risk scores, the 12-lead electrocardiogram, natriuretic peptides, and hand-held echocardiography [196–208]. Uncertainty remains regarding the best test (or combination of tests), how often these tests should be performed, and whether one should entirely focus on high-risk populations (e.g., based upon age, ethnicity, or associated diseases such as vascular disease or diabetes mellitus) rather than broad population screening to maximize cost effectiveness [196, 208–213]. 

Rather than limiting a screening strategy to identify asymptomatic LVSD, another approach is to screen for a number of structural cardiovascular derangements that are associated with an increased risk of developing heart failure including left atrial dilatation, LV diastolic dysfunction, LV hypertrophy, valvular heart disease, and aortic stiffness [200, 205, 214–218]. Indeed, it has been suggested that this would have the potential for greater population impact [218]. However, in the absence of treatment efficacy studies conducted in asymptomatic populations, this approach to population screening should ideally be assessed in a randomized, controlled trial to best define its clinical effectiveness and cost effectiveness [219]. 

## 7. Conclusion

Despite considerable advances in the treatment of heart failure over the last two to three decades, its prevalence continues to rise. Registries and population-based cohorts have revealed substantial gaps and variation in care. Multidisciplinary disease management brings us closer to the models of care that were applied in the randomized, controlled trials that guide our practice. More importantly, these programs improve quality of care, decrease mortality, and are cost effective in reducing hospitalizations in high-risk patients. However, to win the war, we also need to focus on disease prevention to reduce the human and economic burden imposed by heart failure. These strategies include addressing cardiovascular risk factors at a population level and screening for structural cardiovascular derangements such as asymptomatic LVSD.

## Figures and Tables

**Table 1:** Reductions in mortality observed in randomized, controlled trials evaluating drug or device therapies in patients with chronic heart failure that were powered for all-cause mortality and resulted in Level of Evidence A recommendations in clinical guidelines.

Treatment	Target population	Mortality relative risk reduction	Trials
ACE inhibitor*	HF with LVEF ≤40%	16–27%	CONSENSUS [65], SOLVD-T [52]
Beta blocker	HF with LVEF ≤40%	34-35%	CIBIS II [61], MERIT-HF [60], COPERNICUS [59]
Mineralocorticoid receptor antagonist	HF with LVEF ≤35%	24–30%	RALES [64], EMPHASIS HF [62]
Implantable cardioverter defibrillator	HF with LVEF ≤30–35% despite optimal drug therapy	23–31%	MADIT-II [79], SCD-HeFT [78]
Cardiac resynchronization therapy ± implantable cardioverter defibrillator	HF with LVEF ≤30–35% with prolonged QRS duration ≥120–130 ms (especially LBBB) despite optimal drug therapy	24–36%	COMPANION [94], CARE-HF [95], RAFT [92]

*If the patient is unable to tolerate an ACE inhibitor, an angiotensin receptor blocker may be prescribed. ACE inhibitor: angiotensin converting enzyme inhibitor. HF: heart failure. LVEF: left ventricular ejection fraction. LBBB: left bundle branch block. CONSENSUS: cooperative north scandinavian enalapril survival study. SOLVD-T: studies of left ventricular dysfunction-treatment. CIBIS-II: cardiac insufficiency bisoprolol study II. MERIT-HF: metoprolol CR/XL randomised intervention trial in congestive heart failure. COPERNICUS: carvedilol prospective randomized cumulative survival. RALES: randomized aldactone evaluation study. EMPHASIS HF: eplerenone in mild patients hospitalization and survival study in heart failure. MADIT-II: multicenter automatic defibrillator implantation trial II. SCD-HeFT: sudden cardiac death in heart failure trial (SCD-HeFT). COMPANION: comparison of medical therapy, pacing, and defibrillation in heart failure. CARE-HF: cardiac resynchronization in heart failure study. RAFT: resynchronization/defibrillation for ambulatory heart failure trial.

**Table 2:** Studies reporting the proportion of chronic heart failure patients that achieved target doses of beta blockers in “real-world” practice.

Study	Average age (years)	Eligible patients only included*	Patients with documented contraindications or intolerance excluded	Percentage that achieve target dose
Tandon et al. 2004 [137]^a^	69	No	No	18% (24% 1998–2001)
Franciosa et al. 2004 [130]^b^	67	Yes	Yes	27% (primary care) 49% (cardiology)
Mehta et al. 2004 [134]^b^	67	Yes	Yes	7%
Jain et al. 2005 [135]^b^	64	No	No	<25%
Gustafsson et al. 2007 [132]^b^	69	Yes	No	21%
Lainscak et al. 2007 [136]^b^	65	No	Yes	26%
Fonarow et al. 2008 [128]^b^	70	Yes	Yes	8%, 18%
Calvert et al. 2009 [124]^a^	78	No	No	12%
Driscoll et al. 2011 [133]^a^	67	No	No	36% (usual care) 48% (nurse-led)

*Eligibility generally defined as patients with heart failure associated with moderate to severe left ventricular systolic dysfunction.

^a^Proportion taking target dose based upon all patients taking beta blockers.

^b^Proportion taking target dose based upon all patients (including patients taking and patients not taking beta blockers).

**Table 3:** ACCF/AHA and ESC Guidelines for treating asymptomatic LVSD (recommendation; Level of Evidence).

	ACCF/AHA Guidelines 2009 [139]	ESC Guidelines 2005 [48]
ACE inhibitor	(I; A)	(I; A)
Angiotensin receptor blockers	Post-MI and intolerant of ACE I (I; B) No prior MI and intolerant of ACE I (IIa; C)	Post-MI (I; A)
Beta blockers	Recent or remote MI (I; A) No prior MI (I; C)	Post-MI (I; B)
Implantable cardioverter defibrillator	≥40 days post-MI and LVEF ≤30% on optimal medical therapy (IIa; B) Nonischemic cardiomyopathy and LVEF ≤30% on optimal medical therapy (IIb; C)	

ACCF/AHA: American College of Cardiology Foundation/American Heart Association; ESC: European Society of Cardiology; LVSD: left ventricular systolic dysfunction; ACE inhibitor: angiotensin converting enzyme inhibitor; MI: myocardial infarction; LVEF: left ventricular ejection fraction.

Reprinted, with permission, from Atherton, 2012 [140].

## Abbreviations

ACE inhibitors: Angiotensin converting enzyme inhibitors
CRT: Cardiac resynchronization therapy
LV: Left ventricular
LVEF: LV ejection fraction
LVSD: LV systolic dysfunction.
